# Beyond retest gains: episodic memory practice effects and longitudinal cognitive trajectories in cognitively healthy adults

**DOI:** 10.3389/fnagi.2026.1933991

**Published:** 2026-10-12

**Authors:** Simon Fankhauser, Gabriele Cattaneo, Gemma Albi-Villuendas, Javier Solana-Sánchez, Álvaro Pascual-Leone, David Bartrés-Faz

**Affiliations:** 1 Guttmann Hospital de Neurorehabilitació, Badalona, Spain; 2 Departament de Medicina, Facultat de Medicina i Ciències de la Salut, i Institut de Neurociències, Universitat de Barcelona, Barcelona, Spain; 3 Institut de Recerca Germans Trias i Pujol, Badalona, Spain; 4 Institut Universitari de Neurorehabilitació Guttmann adscrit a la Universitat Autònoma de Barcelona, Badalona, Spain; 5 Hinda and Arthur Marcus Institute for Aging Research and Deanna and Sidney Wolk Center for Memory Health, Hebrew SeniorLife, Boston, MA, United States; 6 Department of Neurology, Harvard Medical School, Boston, MA, United States; 7 Institut d'Investigacions Biomèdiques August Pi i Sunyer (IDIBAPS), Barcelona, Spain

**Keywords:** cognitive aging, episodic memory, healthy aging, longitudinal cognition, neuropsychological assessment, practice effects

## Abstract

**Background:**

Practice effects in repeated neuropsychological assessment are usually treated as measurement bias, although they may carry meaningful information. It remains unclear whether individual differences in episodic memory practice effects are associated with cognitive level and longitudinal change beyond generic retest-related gains.

**Methods:**

We analyzed data from 442 cognitively healthy middle-aged and older adults who completed neuropsychological assessments in 2018, 2021, and 2025. Retest-related offsets were modeled explicitly to separate sample-level retest gains from continuous longitudinal change. A baseline-adjusted episodic memory practice effect was derived from change in episodic memory between baseline and first follow-up and included in linear mixed-effects models of global cognition and specific domains, adjusting for age, biological sex, and education.

**Results:**

Retest-related gains were significant at both follow-up waves. After accounting for these offsets, greater episodic memory practice effect was associated with higher global cognitive performance and more favorable longitudinal change. In a robustness analysis using a non-memory global composite, the association with cognitive level remained significant, whereas the association with longitudinal change was attenuated. Domain-specific analyses showed strongest associations for episodic memory, followed by processing speed; reasoning was associated with cognitive level but not change, with no significant associations for attention/working memory or cognitive flexibility.

**Conclusion:**

Episodic memory practice effects appear to carry meaningful information beyond generic retest-related gains. Explicitly modeling retest effects while preserving interindividual differences in practice-related improvement may refine the characterization of longitudinal cognitive trajectories and help identify subtle early differences in cognitive resilience and vulnerability.

## Introduction

Progressive cognitive decline is one of the major concerns associated with mild cognitive impairment and dementia (Knopman and Petersen, 2014), making its accurate measurement and prediction a core objective in research on cognitive aging and neurodegenerative disease. Repeated neuropsychological testing is therefore central for detecting, monitoring, and anticipating disease-related cognitive change (Goldberg et al., 2015; Vivot et al., 2016). This is particularly important because pathological processes may begin years before overt clinical symptoms emerge (Dubois et al., 2016; Jack et al., 2024). However, repeated testing can be difficult to interpret, as performance may improve simply through greater familiarity with the testing procedures or materials (Duff et al., 2010, 2012). These practice effects can be large enough to obscure age-related decline if they are not explicitly accounted for, particularly when cognitive change is evaluated across multiple timepoints (Sanderson-Cimino et al., 2023).

Emerging evidence also suggests that practice effects may be informative rather than merely a source of measurement bias, potentially reflecting individual differences in learning or adaptation to novelty (Suchy et al., 2011). A recent systematic review concluded that smaller or less robust practice effects are consistently associated with poorer current cognitive status, future cognitive decline, and biomarkers of neurodegeneration (Jutten et al., 2020). Similarly, longitudinal studies in healthy middle-aged and older adults have suggested that a reduced improvement across repeated testing may represent an early sign of vulnerability before overt clinical impairment becomes apparent (Jonaitis et al., 2015). Taken together, these findings support the idea that practice effects may capture clinically meaningful variation in risk for subsequent cognitive decline.

A key unresolved issue is how to distinguish average retest-related gains at the sample level from interindividual differences in practice-related improvement that may carry information relevant to later cognitive changes. Observed gains on repeated tests can stem from multiple sources, including familiarity with the procedures, reduced test anxiety, episodic memory for test content, or genuine individual differences in learning responsiveness (Goldberg et al., 2015; Vivot et al., 2016). Short-term gains may also be influenced by baseline performance through regression to the mean and ceiling effects, further complicating the interpretation of raw change scores (Duff et al., 2010; Sánchez-Benavides et al., 2016). At the design level, simulation work has shown that, in conventional longitudinal designs, estimated retest effects may be confounded with age-related and cohort-related change if these components are not modeled appropriately (Hoffman et al., 2011; Sanderson-Cimino et al., 2023). Prior work has therefore emphasized the need to model practice effects explicitly and to account for baseline performance when evaluating their clinical significance (Sánchez-Benavides et al., 2016; Chen et al., 2023).

The present study aims to address this issue in cognitively healthy middle-aged and older adults followed across three waves of neuropsychological assessment. Studying practice effects in this population may be particularly relevant for prevention-oriented cognitive aging research, as subtle differences in learning responsiveness may emerge before overt late-life impairment. We explicitly modeled retest-related offsets at follow-up and separately derived a baseline-adjusted episodic memory practice effect (hereafter memory practice effect) indexing improvement beyond that expected from initial memory level. This approach was intended to distinguish average retest-related shifts at the sample level from interindividual differences in early memory improvement, which may themselves contain meaningful information about cognitive functioning. We then tested whether this memory practice effect, interpreted as a marker of test-related learning responsiveness to initial exposure, was associated with cognitive level and longitudinal change over time. Specifically, we asked whether individual differences in memory practice effect captured meaningful variance beyond sample-level retest-related gains, whether they were associated with global cognitive trajectories, and whether their associations were domain-general or domain-specific. We hypothesized that greater-than-expected improvement in episodic memory from baseline to first retest would be associated with higher overall cognitive performance and more favorable longitudinal change.

## Materials and methods

### Participants

The analytic sample included cognitively healthy adults participating in the prospective, longitudinal Barcelona Brain Health Initiative (BBHI; https://bbhi.cat/en/study). To be eligible for participation, participants had to be between 40 and 65 years old at the start of the study and, as determined by the initial screening, had no history of neurological or psychiatric conditions (Cattaneo et al., 2020). The present analysis included all individuals who completed the full neuropsychological test battery at baseline assessment (2018–2019) and at the two follow-up waves (2020–2022 and 2025). Among 973 participants with a valid baseline cognitive assessment, 442 completed the full neuropsychological battery at all three waves and constituted the present analytic sample. The study was conducted in accordance with the Declaration of Helsinki. All participants provided written informed consent, and the protocol was approved by the Unió Catalana d'Hospitals ethics committee (CEI 18/07 and CEI 22/114).

### Neuropsychological assessment

Participants completed the same standardized neuropsychological battery at baseline and both follow-up waves. The tests were administered in a controlled setting by trained neuropsychologists at Institut Guttmann (Barcelona, Spain), following the published guidelines for the administration and scoring of each instrument. The complete cognitive assessment was conducted in Spanish and lasted approximately 60 min per participant.

The battery included Digit Span forward and backward (Peña-Casanova et al., 2012), Digit Symbol Coding, Cancellation, Matrix Reasoning, and Block Design from the WAIS-IV (Wechsler, 2012), Trail Making Test Parts A and B (Peña-Casanova et al., 2012), and the Spanish version of the Rey Auditory Verbal Learning Test (RAVLT) to assess episodic memory. The version used in the present study was previously validated and normed in the BBHI cohort (Alviarez-Schulze et al., 2022) and comprises a list of 15 unrelated Spanish words presented across five learning trials, followed by delayed recall after approximately 20–25 min and a recognition trial. This Spanish adaptation has also been described in earlier studies by Perea Bartolomé et al. (2000) and Estévez-González et al. (2003).

### Cognitive composite scores

Following previous studies from our group, cognitive composite scores were derived using confirmatory factor analysis (CFA) conducted on baseline neuropsychological data to obtain psychometrically weighted domain measures (Cattaneo et al., 2022). The CFA model was based on a theoretically informed domain structure reflecting established neuropsychological constructs. Trail Making Test Part A was administered but was not retained in the final composite structure as it shares substantial variance with Trail Making Test Part B, making its inclusion less informative within the present domain structure.

Four multi-indicator first-order latent domains were modeled: Attention/working memory (Digit Span forward and backward), processing speed (Digit Symbol Coding and Cancellation), reasoning (Matrix Reasoning and Block Design), and episodic memory (Immediate Recall, Delayed Recall, and Recognition). Cognitive flexibility was modeled as a single-indicator latent variable using Trail Making Test Part B, scored as completion time in seconds, with lower completion times indicating better performance. Scores were reversed for inclusion in the composite so that higher factor scores consistently reflected better cognitive performance.

A second-order global cognition factor was estimated to capture shared variance across domains. Full hierarchical model fit was acceptable (χ^2^ (32) = 91.46, *p* < 0.001; CFI = 0.950; TLI = 0.929; RMSEA = 0.065; SRMR = 0.048). A corresponding first-order model without the higher-order factor showed excellent fit, supporting the adequacy of the domain-level measurement structure (see Supplementary Table 1). Standardized factor scores were extracted and used as cognitive outcomes. To ensure longitudinal comparability, baseline-derived factor loadings were applied to follow-up data.

In a robustness analysis, a non-memory global composite was derived by averaging the standardized factor scores for attention/working memory, processing speed, cognitive flexibility, and reasoning.

### Statistical analysis

A subject-level episodic memory practice effect was derived to quantify interindividual differences in early improvement following initial test exposure. Raw change was calculated as the episodic memory composite score at the first follow-up minus the corresponding baseline score.

$$\begin{array}{l} \left(\Delta Memory_{i}=MemoryW2{,}_{i}-MemoryW1{,}_{i}\right) \end{array}$$

Because the magnitude of observed change may depend on initial performance, including through regression toward the mean and ceiling-related constraints, raw change was regressed on baseline episodic memory performance (Sánchez-Benavides et al., 2016).

$$\begin{array}{l} \left(\Delta Memory_{i}=\alpha +\beta MemoryW1{,}_{i}+\varepsilon_{i}\right) \end{array}$$

The resulting residual (ε_*i*_) therefore represented improvement that was greater or smaller than expected given an individual's initial memory level. Residuals were subsequently standardized, such that higher values indicated greater-than-expected improvement and model coefficients reflected differences per 1-SD increase in this baseline-adjusted measure. The standardized residual was then used as a continuous predictor of cognitive level and longitudinal change.

Importantly, this individual memory practice effect was conceptually distinct from the average retest-related shifts modeled at the sample level. These shifts were represented by separate indicator variables for the first (Wave 2) and second (Wave 3) follow-up assessments, allowing retest-related changes at each follow-up to be distinguished from the underlying longitudinal cognitive trajectory (Vivot et al., 2016; Chen et al., 2023). Thus, the wave indicators were included to separate average retest-related shifts from longitudinal change, whereas the residualized memory practice effect captured between-person differences in early improvement and was used to test whether these differences were associated with cognitive level and longitudinal change.

All analyses were conducted in R (version 4.5.2; R Core Team) using RStudio (version 2026.01.0+392). Longitudinal cognitive trajectories were analyzed using linear mixed-effects models fitted separately for global cognition and each cognitive domain. Fixed effects included time since baseline, retest-related indicators for Wave 2 and Wave 3, baseline age, years of education, biological sex, the memory practice effect, and interactions between time and baseline age, education, and memory practice effect. The same fixed-effects specification, including the Wave 2 and Wave 3 retest-related indicators, was applied to global cognition and all domain-specific models. Random intercepts and random slopes for time were specified at the participant level to account for interindividual differences in baseline cognitive performance and rates of change. For the cognitive flexibility model, the random-slope variance was estimated near zero and produced a singular fit; therefore, a random-intercept-only model was used for this outcome. Models were estimated using restricted maximum likelihood, with Satterthwaite degrees of freedom used for statistical inference. Model diagnostics included visual inspection of residual distributions, Q-Q plots, and residual-vs.-fitted plots, as well as evaluation of random-effects variance components, convergence warnings, and singular model fits. Given that the memory practice effect was a derived predictor, collinearity was additionally evaluated for the memory practice-effect term and its interaction with time using variance inflation factors (VIFs). Model diagnostics and final random-effects structures are summarized in Supplementary Table 2.

To assess whether associations between the memory practice effect and cognitive trajectories were driven solely by episodic memory, a complementary analysis replaced the full global cognition outcome with the non-memory global composite. Model specification was otherwise identical. By excluding episodic memory from the outcome, this analysis reduced direct measurement overlap between the memory-derived predictor and the memory-inclusive global cognition outcome.

As an additional sensitivity analysis, we examined whether the baseline-to-Wave 2 memory practice effect was associated with subsequent Wave 3 cognition after adjustment for the corresponding Wave 2 cognitive score, baseline age, education, sex and the interval in years between Waves 2 and 3. Separate models were fitted for global cognition and the non-memory global composite.

To assess potential selection bias associated with complete three-wave participation, baseline demographic characteristics were compared between participants included in the analytic sample and baseline participants who were not included. Baseline age and years of education were compared using Welch's independent-samples *t*-tests, and biological sex was compared using Pearson's χ^2^ test.

As exploratory moderation analyses, we examined whether baseline age, education or biological sex moderated the association between memory practice effect and cognitive level or longitudinal change. Separate models extended the primary global cognition model by adding the corresponding moderator × memory practice effect and moderator × memory practice effect × time interaction terms. Baseline age and education were mean-centered before constructing the interaction terms. The sex-moderation model additionally included the sex × time interaction to preserve model hierarchy.

## Results

The analytic sample included 442 cognitively healthy adults (mean age = 51.2 years, SD = 5.5; 50.7% women; mean education = 17.2 years, SD = 3.6). Baseline characteristics of the analytic sample and participants not included in the three-wave analysis are presented in Table 1.

**Table 1:** Baseline characteristics of the analytic sample and baseline participants not included in the three-wave analysis.

Characteristics	Analytic sample (*n* = 442)	Not included (*n* = 531)	*p*
Female, *n* (%)	224 (50.7)	249 (46.9)	0.239
Age, years	51.2 (5.5)	54.9 (7.7)	**< 0.001**
Education, years	17.2 (3.6)	16.9 (4.0)	0.211

Values are presented as mean (SD) or n (%), as appropriate. Age and education were compared using Welch's independent-samples *t*-tests and biological sex using Pearson's χ^2^ test. Education data were unavailable for two participants in the non-included group. Bold values indicate statistical significance at *p* < 0.05.

## Baseline comparison of included and non-included participants

Compared with baseline participants who were not included in the three-wave analytic sample (*n* = 531), participants in the analytic sample were younger at baseline (51.2 vs. 54.9 years, *p* < 0.001). No significant group differences were observed in years of education (17.2 vs. 16.9, *p* = 0.211) or biological sex (50.7% vs. 46.9% women, *p* = 0.239).

## Retest-related effects and global cognition

In the global cognition model, both retest-related indicators were significant (Wave 2: β = 0.270, *p* < 0.001; Wave 3: β = 0.363, *p* = 0.017), indicating additional increases in global cognition at follow-up consistent with retest-related practice effects. After accounting for these retest-related effects, interindividual variability in longitudinal trajectories remained. Key model estimates are summarized in Table 2.

**Table 2:** Associations of memory practice effect with global and domain-specific cognitive outcomes.

Outcome	Effect	β	SE	*P*
Global cognition	Wave 2	0.270	0.061	**< 0.001**
	Wave 3	0.363	0.152	**0.017**
	Memory practice effect	0.121	0.036	**< 0.001**
	Memory practice effect × time	0.011	0.004	**0.002**
Global cognition (non-memory)	Wave 2	0.252	0.078	**0.001**
	Wave 3	0.376	0.195	0.055
	Memory practice effect	0.118	0.042	**0.006**
	Memory practice effect × time	0.009	0.005	0.061
Episodic memory	Memory practice effect	0.229	0.046	**< 0.001**
	Memory practice effect × time	0.040	0.006	**< 0.001**
Attention/working memory	Memory practice effect	0.075	0.048	0.117
	Memory practice effect × time	0.011	0.008	0.174
Processing speed	Memory practice effect	0.134	0.063	**0.035**
	Memory practice effect × time	0.014	0.006	**0.023**
Reasoning	Memory practice effect	0.208	0.054	**< 0.001**
	Memory practice effect × time	0.004	0.006	0.495
Cognitive flexibility	Memory practice effect	0.056	0.042	0.177
	Memory practice effect × time	0.006	0.008	0.403

Estimates are fixed effects from linear mixed-effects models. All models included time since baseline, Wave 2 and Wave 3 retest-related indicators, age, education, biological sex, memory practice effect, and interactions of time with age, education, and memory practice effect. For conciseness, Wave 2 and Wave 3 estimates are displayed for the global cognition models only; these indicators were also included in all domain-specific models. The non-memory global composite was included as a robustness analysis. β, fixed-effect estimate; SE, standard error. Bold values indicate statistical significance at *p* < 0.05.

Greater memory practice effect was associated with higher overall global cognitive performance across waves (β = 0.121, *p* = 0.0008) and was significantly associated with estimated longitudinal change in global cognition (β = 0.011 per year, *p* = 0.002). Model-estimated global trajectories at −1 SD, the mean, and +1 SD of the continuous memory practice-effect measure are shown in Figure 1. In the same model, older baseline age was associated with lower global cognitive performance and slightly less favorable change over time, whereas higher education and female sex were associated with higher global cognitive performance.

**Figure 1:**
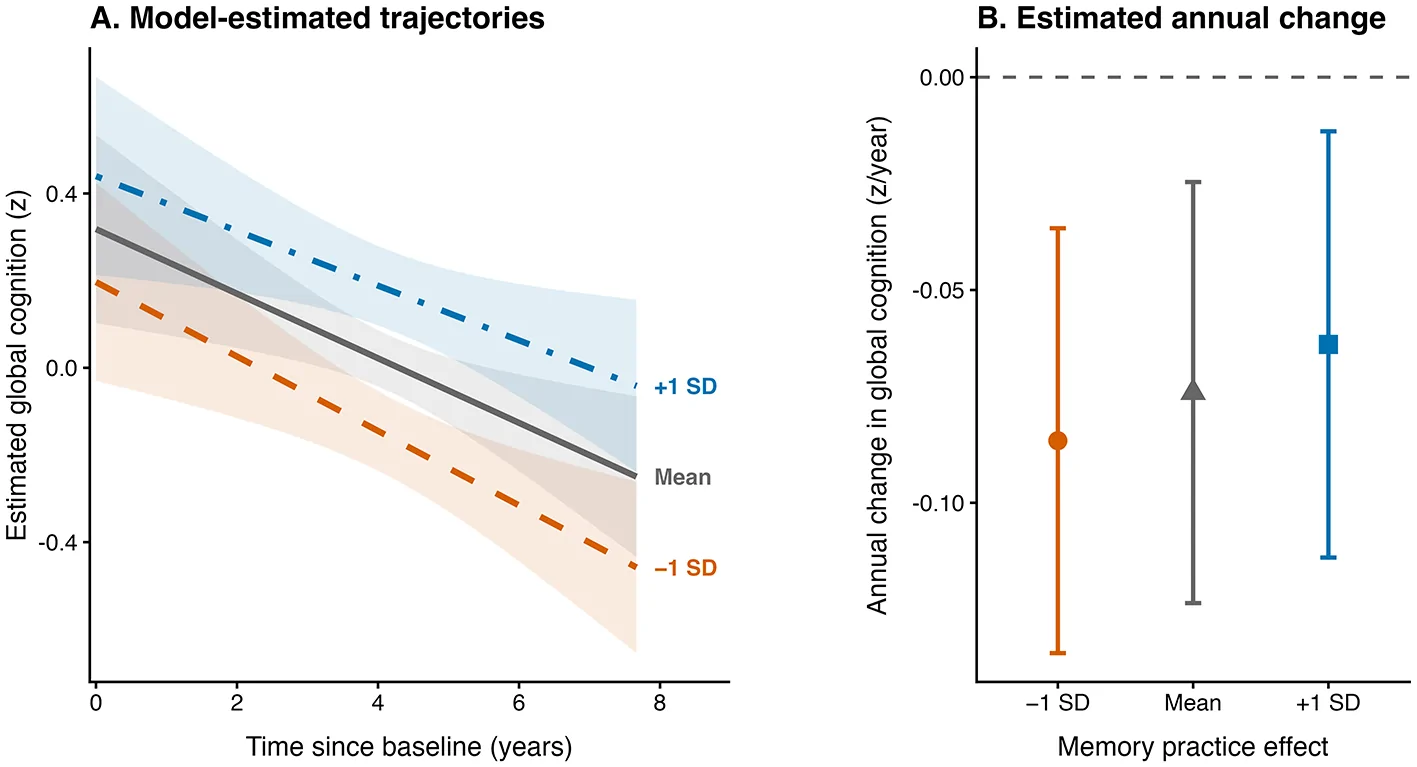
Global cognitive trajectories and annual change by memory practice effect. Panel **(A)** shows model-estimated global cognitive trajectories over time at three representative values of the continuous standardized memory practice-effect measure corresponding to −1 SD, the mean, and + 1 SD, respectively. Shaded areas represent 95% confidence intervals. Line types correspond to these representative values: dashed = −1 SD, solid = mean, and dot-dashed = + 1 SD. Panel **(B)** displays the estimated annual rate of change in global cognition for the same three representative values. These values were selected for visualization only and do not represent participant categories.

In the exploratory age-moderation analysis, baseline age did not significantly moderate the association between memory practice effect and global cognitive level (β = 0.008, *p* = 0.213) or longitudinal change (β < 0.001, *p* = 0.892). Similarly, baseline education did not significantly moderate the association between memory practice effect and global cognitive level (β = 0.005, p =0.593) or longitudinal change (β = −0.001, *p* = 0.376). Biological sex likewise did not moderate the association between memory practice effect and global cognitive level (β = −0.038, *p* = 0.580) or longitudinal change (β = −0.004, *p* = 0.563; Supplementary Table 4).

## Robustness analysis using non-memory composite score

In the robustness analysis using the non-memory global composite, the association with overall cognitive level remained significant (β = 0.118, *p* = 0.006), whereas the interaction with time was attenuated and no longer conventionally significant (β = 0.009 per year, *p* = 0.061).

## Collinearity and sensitivity analyses

Collinearity diagnostics provided no evidence that the derived memory practice-effect predictor introduced problematic multicollinearity; VIFs were 1.23 for the memory practice-effect term and 1.22 for its interaction with time. Consistent with its residualized construction, the memory practice effect was also essentially uncorrelated with baseline episodic memory performance (*r* < 0.001).

In the temporally separated sensitivity analysis (*N* = 442), memory practice effect was not significantly associated with Wave 3 global cognition after adjustment for Wave 2 global cognition and covariates (β = −0.040, SE = 0.021, *p* = 0.052). Similarly, there was no association with Wave 3 non-memory cognition after adjustment for Wave 2 non-memory cognition (β = −0.008, SE = 0.026, *p* = 0.771). Full estimates from these sensitivity analyses are provided in Supplementary Table 3.

## Domain-specific associations

Domain-specific analyses showed the strongest associations for episodic memory, for which memory practice effect was associated with both cognitive level (β = 0.229, *p* < 0.001) and longitudinal change (β = 0.040, *p* < 0.001). Significant associations with both level (β = 0.134, *p* = 0.035) and change (β = 0.014, *p* = 0.023) were also observed for processing speed. For reasoning, memory practice effect was associated with higher cognitive level (β = 0.208, *p* < 0.001) but not with longitudinal change (β = 0.004, *p* = 0.495). No significant associations were observed for attention/working memory or cognitive flexibility.

Overall, significant associations were strongest for episodic memory and global cognition, more limited for processing speed and reasoning, and absent for attention/working memory and cognitive flexibility.

## Discussion

The present study examined whether individual differences in episodic memory practice effects, adjusted for baseline performance, were associated with cognitive level and longitudinal change in cognitively healthy middle-aged and older adults. By explicitly separating sample-level retest-related gains from interindividual differences in practice-related improvement, we aimed to determine whether early improvement after test exposure may capture variation not fully attributable to measurement artifact. Overall, three main findings emerged. First, retest-related gains were substantial and detectable at both follow-up waves, underscoring the importance of explicitly modeling these effects in longitudinal cognitive analyses. Second, accounting for generic retest-related variance did not eliminate between-person variability in cognitive trajectories, indicating that interindividual differences in longitudinal change remained after separating retest-related gains from continuous change over time. Third, memory practice effects were associated with higher global cognitive performance across waves and, particularly for memory-inclusive outcomes, with more favorable estimated change over time.

The finding of substantial retest-related gains at both follow-up waves is consistent with a large body of work showing that repeated exposure to neuropsychological tests can produce systematic score improvements that are not attributable to true cognitive change (Calamia et al., 2012). Importantly, in the present study these effects were detectable despite the multi-year intervals between assessments, underscoring that retest-related gains are not limited to short retest intervals. Prior reviews have emphasized that such effects are both pervasive and large enough to distort longitudinal inference, arguing that explicit handling of practice effects should be considered a standard requirement in studies of cognitive aging (Sanderson-Cimino et al., 2023; Freedman and Hu, 2024). Our results reinforce this point by showing that retest-related gains were clearly present at both follow-up assessments and therefore should not be treated as negligible background noise. Accounting for these generic retest-related gains, however, did not remove variability in cognitive trajectories across individuals. This is important because it indicates that longitudinal heterogeneity is not reducible to repeated-testing artifacts alone but instead reflects meaningful between-person differences in cognitive evolution that remain observable once retest-related variance is modeled explicitly.

After adjustment for baseline performance, memory practice effects were associated with both higher global cognitive performance and more favorable change over time. Individuals who showed greater-than-expected improvement in episodic memory from baseline to the first follow-up exhibited better overall cognitive performance across all measurement waves and less unfavorable estimated trajectories over time. These findings extend prior work showing that episodic memory practice effects may carry information potentially relevant to cognitive characterization (Duff et al., 2007; Machulda et al., 2013; Jutten et al., 2020) by showing that this association remains observable even after generic retest-related gains are modeled explicitly.

Accordingly, the informative value of early episodic memory improvement is not fully reducible to sample-level retest offsets alone. Given that the memory practice effect was derived from early change in episodic memory, this finding is better interpreted as an informative early longitudinal signal than as evidence of an independent prognostic marker. One possible explanation is that greater improvement in episodic memory may reflect individual differences in learning efficiency, test-related adaptation, or reserve-related processes that are relevant to cognitive functioning over time (Pettigrew and Soldan, 2019). Individuals who benefit more from an initial exposure to memory testing may be better able to encode, consolidate, and retrieve information efficiently, and this capacity may in turn be associated with more favorable global cognitive trajectories.

Conversely, reduced benefit from repeated testing may reflect early vulnerability, including subtle difficulties in encoding or consolidation that are not yet fully visible in standard longitudinal scores. This interpretation is broadly consistent with work linking attenuated practice effects to poorer current cognitive status, future decline, amyloid pathology, and conversion risk in cognitively unimpaired and clinically vulnerable older adults (Duff et al., 2015; De Simone et al., 2021; Zheng et al., 2022). A more specific example comes from recent work showing that repeated learning over days can reveal amyloid-related memory differences that are not apparent on conventional single-timepoint assessment, suggesting that altered memory consolidation may be one mechanism linking diminished practice effects to preclinical Alzheimer's disease (Papp et al., 2024). However, given the observational design and the task-specific derivation of the memory practice effect, the present data do not allow direct inference regarding the mechanisms underlying these associations.

Results of the robustness analysis suggest that the observed associations were not solely driven by overlap between the episodic memory predictor and the memory-inclusive global cognitive outcome. When global cognition was recalculated excluding episodic memory tests, the association with overall cognitive level remained significant, whereas the association with longitudinal change was attenuated and no longer conventionally significant. The persistence of the association with cognitive level is consistent with evidence that cognitive domains are partly organized around a common general factor and that changes in one domain may covary with changes in others, including both fluid and crystallized abilities (Tucker-Drob et al., 2022). That said, the attenuation of the longitudinal association indicates that the practice-effect signal may be most sensitive to outcomes that include episodic memory, which is plausible given the central role of episodic memory in cognitive aging and neurodegenerative disease (Tromp et al., 2015). Taken together, these findings suggest that the memory practice effect may contain information extending beyond the memory domain to some extent, while remaining especially relevant for outcomes that are sensitive to memory-related performance.

Domain-specific analyses similarly pointed to a selective rather than domain-general pattern of associations. The strongest effects were observed for episodic memory, followed by processing speed, whereas reasoning was associated with higher cognitive level but not with longitudinal change, and no significant associations were observed for attention/working memory or cognitive flexibility. This selective pattern suggests that the interpretive value of practice effects may vary across cognitive domains rather than reflecting a uniform property of repeated testing, consistent with evidence that retest effects may differ across episodic memory and executive-function measures (Glisky et al., 2022). A possible interpretation is that the domains most strongly associated with later outcomes are those more closely related to learning efficiency, adaptive responding, or early vulnerability to age-related change. Alternatively, because the practice effect predictor was derived from episodic memory, stronger associations for memory-related outcomes are also expected on methodological grounds and should therefore be interpreted cautiously. More broadly, these findings argue against treating practice effects as a single undifferentiated phenomenon and instead suggest that their interpretive value may depend on the cognitive system from which they are derived.

From a methodological perspective, one of the main contributions of the present study is the explicit distinction between sample-level retest-related gains and interindividual differences in practice-related improvement. These components are often conflated in longitudinal cognitive research, where practice effects are either treated exclusively as nuisance variance or interpreted descriptively without being separated from generic retest-related change. Our findings suggest that these perspectives should not be viewed as mutually exclusive. Generic retest effects can and should be modeled to reduce bias in estimates of longitudinal change, while individual differences in episodic memory improvement may still contain potentially informative variation. Treating all practice effects as nuisance variance, or removing them entirely from cognitive outcomes, may therefore obscure variation that is potentially informative rather than purely artifactual. This issue is not only methodological but also clinically relevant, as prior work has shown that failure to adjust for practice effects can contribute to underdiagnosis of mild cognitive impairment and delay recognition of clinically meaningful change (Elman et al., 2018; Sanderson-Cimino et al., 2022). From a clinical perspective, an important next question is whether incorporating individual practice effects improves the early identification of emerging cognitive impairment beyond conventional cognitive scores. Although the present study cannot determine whether incorporating individual practice effects would identify emerging cognitive impairment earlier than conventional cognitive scores or quantify such a temporal advantage, the observed associations support evaluating this possibility in longitudinal cohorts with prospectively ascertained cognitive impairment. Overall, the present findings support a more differentiated view in which practice effects represent both a methodological challenge and a potentially informative marker of test-related change, while also requiring cautious interpretation regarding mechanism and prognostic independence.

## Limitations

This study has several limitations. First, the sample consisted of cognitively healthy and relatively well-educated individuals (78.5% with a university degree) and covered a comparatively narrow age range, which may restrict generalizability to more diverse, clinically vulnerable, or substantially older populations. In particular, higher education and preserved cognitive functioning may be associated with greater cognitive reserve, higher baseline performance, and potentially different magnitudes or patterns of practice effects than would be observed in more heterogeneous or clinically vulnerable samples. Exploratory moderation analyses provided no evidence that baseline age or education modified the association between memory practice effect and either global cognitive level or longitudinal change; however, the relatively restricted distributions of these characteristics may have limited our ability to detect such effect modification. Biological sex likewise did not moderate the association between memory practice effect and either global cognitive level or longitudinal change. Nevertheless, the present cohort does not allow us to determine whether sex-related patterns in practice effects or their association with cognitive trajectories are similar at substantially older ages, when dementia risk is higher. These null moderation findings should therefore be interpreted within the demographic range represented in the present cohort and should not be assumed to apply equally to substantially older or more heterogeneous populations. At the same time, this sample provided a valuable context for examining the potential interpretive relevance of practice effects in a high-functioning group, where subtle individual differences may be especially informative because overt impairment is not yet present.

Second, because the study was observational, the mechanisms underlying individual differences in practice effects cannot be established directly. This is particularly relevant for the interpretation of our main finding, namely that greater improvement in episodic memory was associated with higher cognitive level and more favorable longitudinal change. Practice effects are thought to reflect multiple partly overlapping processes, including learning efficiency, strategy acquisition, test familiarity, motivation, and possibly early neuropathological vulnerability, all of which have been discussed in the broader literature on repeated testing and cognitive reserve. Accordingly, although our results suggest that episodic memory practice effects may carry information relevant to cognitive characterization, they do not allow us to determine which of these mechanisms primarily drives the observed association.

Third, the episodic memory practice effect was derived from a specific task and may therefore not generalize to other memory measures or paradigms. As a result, the associations observed in this study should not be assumed to extend automatically to episodic memory more broadly. Even so, the use of a clearly defined and consistently administered task provided a focused and interpretable basis for evaluating the study hypothesis. In addition, the cognitive flexibility model did not support estimation of participant-specific slopes, and results for this domain should therefore be interpreted cautiously.

Fourth, analyses were restricted to participants who completed the full neuropsychological battery at all three waves. This complete-case approach may have introduced selection bias. Compared with baseline participants who were not included in the three-wave analytic sample, included participants were younger, whereas no significant differences were observed in education or biological sex. Nevertheless, differences in baseline cognitive performance or other characteristics not examined in the present comparison cannot be excluded. Accordingly, some degree of selection bias may remain, and the younger age of the retained sample may limit the generalizability of the findings to older individuals from the original baseline cohort.

Fifth, although baseline-derived factor loadings were applied to follow-up data to improve comparability of the cognitive composites, formal longitudinal measurement invariance was not tested. Future work should evaluate whether the measurement structure remains stable across repeated assessments.

Sixth, although three assessment waves offered a meaningful longitudinal window, longer follow-up will be important to more fully capture cognitive trajectories and to determine whether these early differences are related to later clinical outcomes. Practice effects may be particularly relevant in the preclinical or very early stages of decline, whereas the consequences for clinically meaningful endpoints such as persistent impairment, mild cognitive impairment, or dementia may only become apparent over a longer time span. The present study is therefore informative with respect to early longitudinal patterns but does not yet establish how far these associations extend into later disease progression.

Finally, a key limitation is that the memory practice effect was derived from change between baseline and Wave 2, while these same assessments also contributed to the primary longitudinal outcomes, resulting in shared measurement information and the possibility of partial mathematical coupling. Residualization removed the linear dependence of the practice-effect score on baseline memory performance, and the association with cognitive level remained when episodic memory was excluded from the outcome. However, memory practice effect did not explain additional Wave 3 global or non-memory cognitive performance after accounting for the corresponding Wave 2 cognitive level. These findings support a cautious interpretation of the memory practice effect as an early longitudinal correlate rather than an independent prognostic marker. A related consideration is that the same episodic memory materials were repeated across waves. This may increase sensitivity to individual differences in the ability to benefit from previous test exposure, which is central to the practice-effect construct examined here, but it may also facilitate later performance through memory for specific items and thereby partially obscure underlying decline. Although the wave-specific retest terms account for average follow-up-related shifts, they cannot distinguish item-specific familiarity from procedural familiarity, strategy acquisition, or broader learning-related responsiveness at the individual level. The persistence of the association with cognitive level when episodic memory was excluded from the global outcome suggests that the observed relationship was not entirely confined to performance on the repeated memory material; however, the weaker longitudinal association in the non-memory composite requires careful interpretation, and future studies that compare identical, recurring materials with well-matched alternative forms are encouraged.

## Future research

Given the limitations outlined above, future studies should examine whether these findings generalize to older, more heterogeneous and clinically enriched samples, including individuals with subjective cognitive decline, mild cognitive impairment, or biomarker evidence of neurodegenerative disease. Although exploratory analyses did not indicate that age, education, or biological sex moderated the association between the memory practice effect and cognition within the present sample, broader demographic ranges will be needed to determine whether these null moderation findings are robust beyond the present cohort. Importantly, clinically enriched longitudinal cohorts with sufficient incident cognitive impairment will also be needed to establish whether practice effects provide incremental prognostic information beyond current cognitive performance, identify clinically meaningful thresholds, and determine whether they can signal emerging impairment earlier than conventional cognitive scores. Integrating such longitudinal cognitive data with neuroimaging and biomarkers may further clarify whether reduced practice effects reflect early neuropathological processes, diminished neurocognitive plasticity, or both. Extending this framework to other cognitive domains, testing paradigms, and dynamic modeling approaches may also improve our understanding of when and for whom practice effects are informative (Bender et al., 2022).

## Conclusion

In conclusion, the present findings indicate that episodic memory practice effects may capture variation not fully attributable to generic retest-related gains. Although retest effects were substantial, they did not explain all variability in longitudinal cognitive performance. Individuals with greater-than-expected improvement in episodic memory after initial test exposure showed better overall cognitive functioning and more favorable estimated trajectories over time, particularly when outcomes included memory performance. This suggests that practice effects may reflect individual differences associated with later cognitive performance and estimated change. These findings support the view that practice effects are not only a methodological challenge but also a potentially informative marker of test-related change in longitudinal cognitive assessment. Accordingly, explicitly modeling retest effects is important for reliably characterizing cognitive trajectories, since failing to do so may mask meaningful change and obscure early signs of emerging decline; at the same time, preserving individual differences in practice-related improvement may help describe subtle variation in cognitive aging before overt impairment is present.

## Data Availability

The data supporting the findings of this study are not publicly available due to ethical and privacy restrictions related to human participant data. Data may be made available from the corresponding author upon reasonable request and subject to approval by the relevant ethics and data-governance procedures.
