# Supplementary material for: Beyond retest gains: episodic memory practice effects and longitudinal cognitive trajectories in cognitively healthy adults
Source: Front Aging Neurosci. 2026 Oct 12;18:1933991. doi: 10.3389/fnagi.2026.1933991 (PMC13696485; doi:10.3389/fnagi.2026.1933991)
Supplement: Supplementary file 1 [file Data_Sheet_1.docx]

**Supplementary Material**

**Supplementary Table S1.** Confirmatory factor analysis of cognitive domains and global cognition at baseline.

Confirmatory factor analyses were conducted on baseline neuropsychological data to derive cognitive composite scores. A theoretically informed first-order CFA model was first used to evaluate the domain-level measurement structure. This model included four multi-indicator latent domains: Attention/working memory, processing speed, reasoning, and episodic memory, together with a single-indicator cognitive flexibility factor defined by reverse-scored Trail Making Test Part B. The first-order model showed excellent fit to the data (χ²(27) = 48.33, p = .007; CFI = 0.981; TLI = 0.968; RMSEA = 0.045, 90% CI [0.023, 0.065]; SRMR = 0.029).

A second-order hierarchical CFA model was then estimated to derive a global cognition factor capturing shared variance across the cognitive domains. This model showed acceptable fit to the data (χ²(32) = 91.46, p < .001; CFI = 0.950; TLI = 0.929; RMSEA = 0.065, 90% CI [0.049, 0.081]; SRMR = 0.048). Standardized first-order and second-order loadings are shown below.

**Panel A.** Model Fit

| **Model** | **N** | **χ²** | **df** | **p** | **CFI** | **TLI** | **RMSEA** | **RMSEA 90% CI** |
| --- | --- | --- | --- | --- | --- | --- | --- | --- |
| First-order CFA | 442 | 48.33 | 27 | 0.007 | 0.981 | 0.968 | 0.045 | [0.023, 0.065] |
| Hierarchical second-order CFA | 442 | 91.46 | 32 | <.001 | 0.950 | 0.929 | 0.065 | [0.049, 0.081] |

**Panel B.** Standardized first-order factor loadings

| **Latent domain** | **Indicator** | **Standardized loading** |
| --- | --- | --- |
| Attention/Working Memory | Digit Span Forward | 0.458 |
| Attention/Working Memory | Digit Span Backward | 0.852 |
| Processing Speed | Digit Symbol Coding | 0.775 |
| Processing Speed | Cancellation | 0.550 |
| Cognitive Flexibility | TMT-B (reverse scored) | Fixed |
| Reasoning | Matrix Reasoning | 0.662 |
| Reasoning | Block Design | 0.685 |
| Episodic Memory | Immediate Recall | 0.848 |
| Episodic Memory | Delayed Recall | 0.924 |
| Episodic Memory | Recognition | 0.600 |

**Panel C.** Standardized second-order loadings on global cognition

| **Higher-order factor** | **First-order domain** | **Standardized loading** |
| --- | --- | --- |
| Global Cognition | Attention/Working Memory | 0.564 |
| Global Cognition | Processing Speed | 0.808 |
| Global Cognition | Cognitive Flexibility | 0.458 |
| Global Cognition | Reasoning | 0.740 |
| Global Cognition | Episodic Memory | 0.509 |

**Note.** Cognitive flexibility was modeled as a single-indicator latent factor defined by reverse-scored Trail Making Test Part B; its loading was fixed for model identification and should not be interpreted as an estimated standardized loading. CFI = comparative fit index; TLI = Tucker–Lewis index; RMSEA = root mean square error of approximation; SRMR = standardized root mean square residual.

**Supplementary Table S2.** Model diagnostics and random-effects structure.

| **Model** | **Random-effects structure** | **Singular fit** | **Convergence warning** | **Residual SD** | **REML criterion** |
| --- | --- | --- | --- | --- | --- |
| Global cognition | Random intercept + random slope for time | No | No | 0.292 | 1929.0 |
| Non-memory Global Cognition | Random intercept + random slope for time | No | No | 0.397 | 2546.1 |
| Attention/Working memory | Random intercept + random slope for time | No | No | 0.716 | 3578.6 |
| Processing speed | Random intercept + random slope for time | No | No | 0.506 | 3378.5 |
| Cognitive Flexibility | Random intercept only | No | No | 0.707 | 3437.6 |
| Reasoning | Random intercept + random slope for time | No | No | 0.481 | 3127.9 |
| Episodic Memory | Random intercept + random slope for time | No | No | 0.497 | 2979.1 |

**Note.** All final models showed no convergence warnings or singular fits. The cognitive flexibility model was estimated with a random intercept only because the initial random-intercept plus random-slope model produced a singular fit due to near-zero random-slope variance. REML = restricted maximum likelihood.

**Supplementary Table S3.** Temporally separated sensitivity analyses of Wave 3 cognition.

| **Predictor** | **Wave 3 global cognition, Estimate (SE)** | | **95% CI** | **p** | **Wave 3 non-memory cognition, Estimate (SE)** | **95% CI** | **p** |
| --- | --- | --- | --- | --- | --- | --- | --- |
| Corresponding Wave 2 cognitive score | | 0.811 (0.027) | [0.758, 0.864] | **<.001** | 0.739 (0.029) | [0.683, 0.795] | **<.001** |
| Memory practice effect | −0.040 (0.021) | | [−0.081, 0.0003] | .052 | −0.008 (0.026) | [−0.058, 0.043] | .771 |
| Baseline age | −0.018 (0.004) | | [−0.025, −0.010] | **<.001** | −0.022 (0.005) | [−0.032, −0.013] | **<.001** |
| Education | 0.006 (0.006) | | [−0.005, 0.017] | .296 | 0.010 (0.007) | [−0.004, 0.024] | .156 |
| Sex: Woman vs Man | 0.050 (0.039) | | [−0.027, 0.127] | .204 | 0.018 (0.050) | [−0.081, 0.117] | .716 |
| Wave 2–Wave 3 interval (years) | −0.121 (0.035) | | [−0.191, −0.052] | **<.001** | −0.134 (0.046) | [−0.224, −0.045] | **.003** |

**Model information:** N = 442 for both models. Global cognition model: R² = .748, adjusted R² = .744. Non-memory cognition model: R² = .686, adjusted R² = .682.

**Note.** Memory practice effect was derived from baseline-to-Wave 2 episodic-memory change after adjustment for baseline episodic-memory performance and subsequent standardization. Each model examined Wave 3 cognitive performance while adjusting for the corresponding Wave 2 cognitive score, baseline age, baseline education, baseline biological sex, and the interval in years between Waves 2 and 3. The non-memory composite excluded episodic memory.

**Supplementary Table S4.** Exploratory moderation analyses of the association between memory practice effect and global cognition.

| **Moderator** | **Interaction** | **β** | **SE** | **95% CI** | **p** |
| --- | --- | --- | --- | --- | --- |
| **Baseline age** | Age × memory practice effect | 0.0079 | 0.0063 | [−0.0046, 0.0203] | .213 |
|  | Age × memory practice effect × time | 0.00009 | 0.00065 | [−0.00120, 0.00137] | .892 |
| **Baseline education** | Education × memory practice effect | 0.0052 | 0.0097 | [−0.0139, 0.0242] | .593 |
|  | Education × memory practice effect × time | −0.00088 | 0.00099 | [−0.00282, 0.00107] | .376 |
| **Biological sex** | Sex × memory practice effect | −0.0379 | 0.0683 | [−0.172, 0.0964] | .580 |
|  | Sex × memory practice effect × time | −0.00432 | 0.00747 | [−0.0190, 0.0104] | .563 |

**Note.** Separate exploratory linear mixed-effects models examined whether baseline age, baseline education, or biological sex moderated the association between memory practice effect and global cognitive level and longitudinal change. Age and education were mean-centered before constructing interaction terms. For the sex-moderation model, a sex × time term was additionally included to preserve model hierarchy. Men were the reference category for biological sex. The two-way moderator × memory practice-effect interaction tests moderation of the association with global cognitive level, whereas the three-way moderator × memory practice effect × time interaction tests moderation of longitudinal change. Models otherwise followed the fixed- and random-effects structure of the primary global cognition model.
